# Outdoor social distancing behaviors changed during a pandemic: A longitudinal analysis using street view imagery

**DOI:** 10.1371/journal.pone.0315132

**Published:** 2024-12-05

**Authors:** Matthew Martell, Chris Salazar, Nicole A. Errett, Scott B. Miles, Joseph Wartman, John Y. Choe

**Affiliations:** 1 Industrial & Systems Engineering, University of Washington, Seattle, WA, United States of America; 2 Environmental & Occupational Health Sciences, University of Washington, Seattle, WA, United States of America; 3 Human Centered Design & Engineering, University of Washington, Seattle, WA, United States of America; 4 Civil & Environmental Engineering, University of Washington, Seattle, WA, United States of America; Shimane Daigaku, JAPAN

## Abstract

Social distancing, defined as maintaining a minimum interpersonal distance (often 6 ft or 1.83 m), is a non-pharmaceutical intervention to reduce infectious disease transmission. While numerous quantitative studies have examined people’s social distancing behaviors using mobile phone data, large-scale quantitative analyses of adherence to suggested minimum interpersonal distances are lacking. We analyzed pedestrians’ social distancing behaviors of using 3 years of street view imagery collected in a metropolitan city (Seattle, WA, USA) during the COVID-19 pandemic. We employed computer vision techniques to locate pedestrians in images, and a geometry-based algorithm to estimate physical distance between them. Our results indicate that social distancing behaviors correlated with key factors such as vaccine availability, seasonality, and local socioeconomic data. We also identified behavioral differences at various points of interest within the city (e.g., parks, schools, faith-based organizations, museums). This work represents a first of its kind longitudinal study of outdoor social distancing behaviors using computer vision. Our findings provide key insights for policymakers to understand and mitigate infectious disease transmission risks in outdoor environments.

## Introduction

The COVID-19 pandemic prompted unprecedented responses from national and local governments worldwide, including emergency declarations and the implementation of social distancing and mask-wearing measures to mitigate virus transmission [1]. In the United States, following the first confirmed case in Washington state in January 2020 [2], the federal government swiftly enacted restrictions. By March 2020, gatherings of more than 10 people were discouraged, and remote work was strongly advised. Concurrently, Washington state imposed more stringent measures, including the closure of schools, dine-in restaurants, and entertainment venues, coupled with a statewide stay-at-home order. The state later mandated face masks in public spaces in June 2020. The United States Centers for Disease Control and Prevention (CDC) recommended a physical distance of at least six ft both indoors and outdoors to prevent the spread of the virus. During the early days of the pandemic, some of the largest superspreading events in the US occurred when these recommendations were violated by large groups of people [3]. The CDC recommendations were not relaxed until August 2022, although most states officially ‘reopened’ well before then, including Washington State on June 30, 2021.

While it is known that it is easier for the disease to spread indoors when compared to outdoors, there are still many examples of outdoor transmission occurring, sometimes at a large scale [4–6]. Studies that have attempted to understand how outdoor transmission occurs and what factors reduce transmission risk showed that there is a strong risk of outdoor transmission under the right circumstances. Hang et al. [7] studied outdoor transmission in a street canyon environment and showed that outdoor transmission risk is highest in downwind areas. Fan et al. [8] likewise studied outdoor transmission in a street canyon environment using simulation methods. They recommended an outdoor distance of 4 meters when relative humidity and winds are low. Lastly, Kia et al. [9] studied outdoor transmission risk on the University of Houston campus, focusing on areas where air is not quickly ventilated. They showed that there were ‘hot spots’ on campus where air could take as long as 1,000 seconds to ventilate. Given the risk of outdoor transmission, it is useful to understand the public’s outdoor social distancing behaviors during the pandemic. This can help governments craft targeted policies based on known information to prevent the spread of future infectious diseases.

Though many agencies worldwide issued similar recommendations regarding preventative measures against COVID-19 transmission, compliance has varied, as evidenced by observational studies. For example, Rahimi et al. [10, 11] conducted a comprehensive assessment of the impact of socioeconomic status on face mask usage among pedestrians during the pandemic in Ahvaz, Iran. Additionally, Davis and Esposito [12] revealed that social disparities, including income, education, and race, significantly reduce social distancing behaviors in diverse and divided communities. These observations have spurred further research, such as the study by Hoeben et al. [13], which used CCTV (closed-circuit television) footage from the Netherlands to demonstrate that adherence to the 1.5-meter distancing guideline initially met compliance but diminished over time. This variability in adherence has prompted researchers to explore various aspects of public compliance with social distancing measures.

Additionally, there are many studies that analyze community mobility and congregating behaviors using mobile phone data or self-reported survey data [1, 14–18]. One finding from these studies is that pedestrians were less likely to strictly follow COVID-19 risk-reducing behaviors, including avoiding congregating after getting vaccinated [18, 19]. Another is that there are gaps in social distancing behaviors between different socioeconomic and racial groups [14]. While the findings of the above studies are extremely useful for understanding people’s congregating behaviors, there is a gap in understanding how people are adhering to interpersonal distance recommendations. Instead of measuring physical distances, these studies typically use foot traffic at specific locations of interest, and survey responses on social distancing behaviors as measures of adherence. While efforts are made to avoid it, there are known representation issues associated with mobility data captured by cell phones [20, 21], and sampling bias associated with surveys.

While the aforementioned research utilized indirect measurements to study social distancing behavior, other studies have employed more direct methods. For example, Seres et al. [22] analyzed how face mask compliance influenced adherence to social distancing in queues. Although insightful, studies like these and the CCTV analysis by Hoeben et al. [13] capture only momentary behaviors, not long-term patterns. To overcome this limitation, new deep learning models have been developed. These large-scale models not only classify pedestrians but also estimate social distancing [23, 24], providing valuable tools for social scientists as outlined by Bernasco et al. [25]. However, these methods have primarily been applied in CCTV settings over a study period of months. CCTV data, while valuable, is location-limited, and requires permission to either access data, or install a camera. Additional insights could be gained from a data set providing greater coverage of an area of interest over a longer period of time.

**Fig 1:**
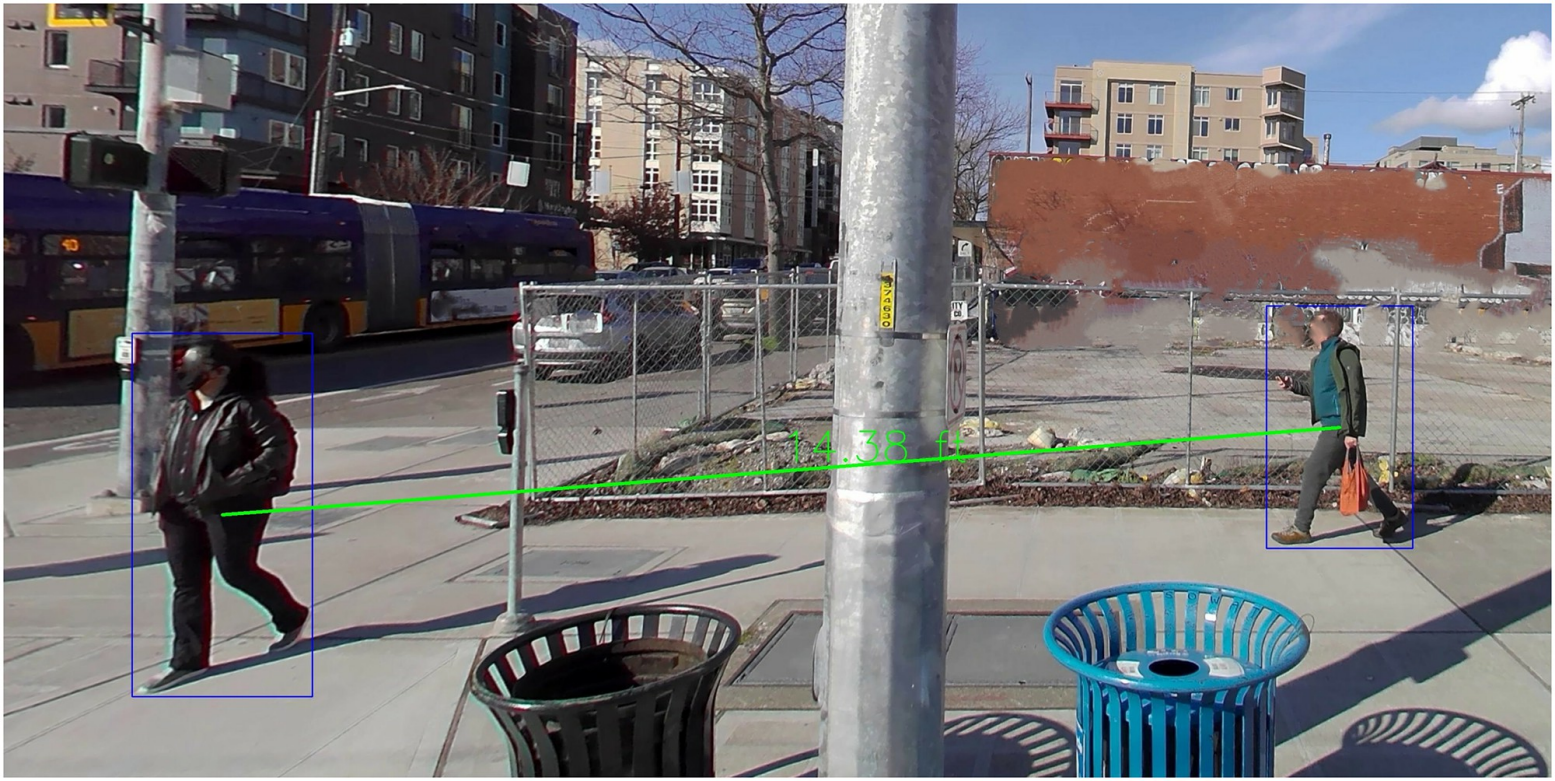
Sample output of the pedestrian detection and social distance estimation algorithm. There are 2 pedestrians, with an estimated 14.38 ft between them. Additional sample images are available in the S1 File.

In this study, the large-scale, longitudinal data set of street-view imagery captured in the city of Seattle, as part of the study described in Martell et al. [26], is used to track trends in social distancing over time. Physical distance between pedestrians is estimated using an algorithmic approach [27]. The end result of this process is a set of images, with the pedestrians identified, and the distances between them estimated, as seen in Fig 1, allowing for an empirical analysis of social distancing behaviors. The data set used in this study extends from May 2020 through July 2023. It can be used to track overall trends in community mobility, in addition to social distancing, using the estimated distances generated from the street view imagery. Our main contribution is a first of its kind method for empirically studying longitudinal outdoor social distancing behaviors. Given the geospatial nature of the data, this method allows for subsets of the data at specific locations of interest to be studied. In turn, we can compare different geographic areas such as census tracts to understand the different drivers of social distancing behaviors. Secondary contributions include empirical confirmation of results from qualitative studies on social distancing behaviors, and additional perspective on COVID-19 related inequities in the US.

## Methods

This section describes the methods used, including data collection, and statistical methods. The original data set in this work is featured in Martell et al. [26]. We do not describe the process for the generation of that data set here, but rather focus on the new features we added, most notably the physical distance estimates.

### Data description and processing

**Fig 2:**
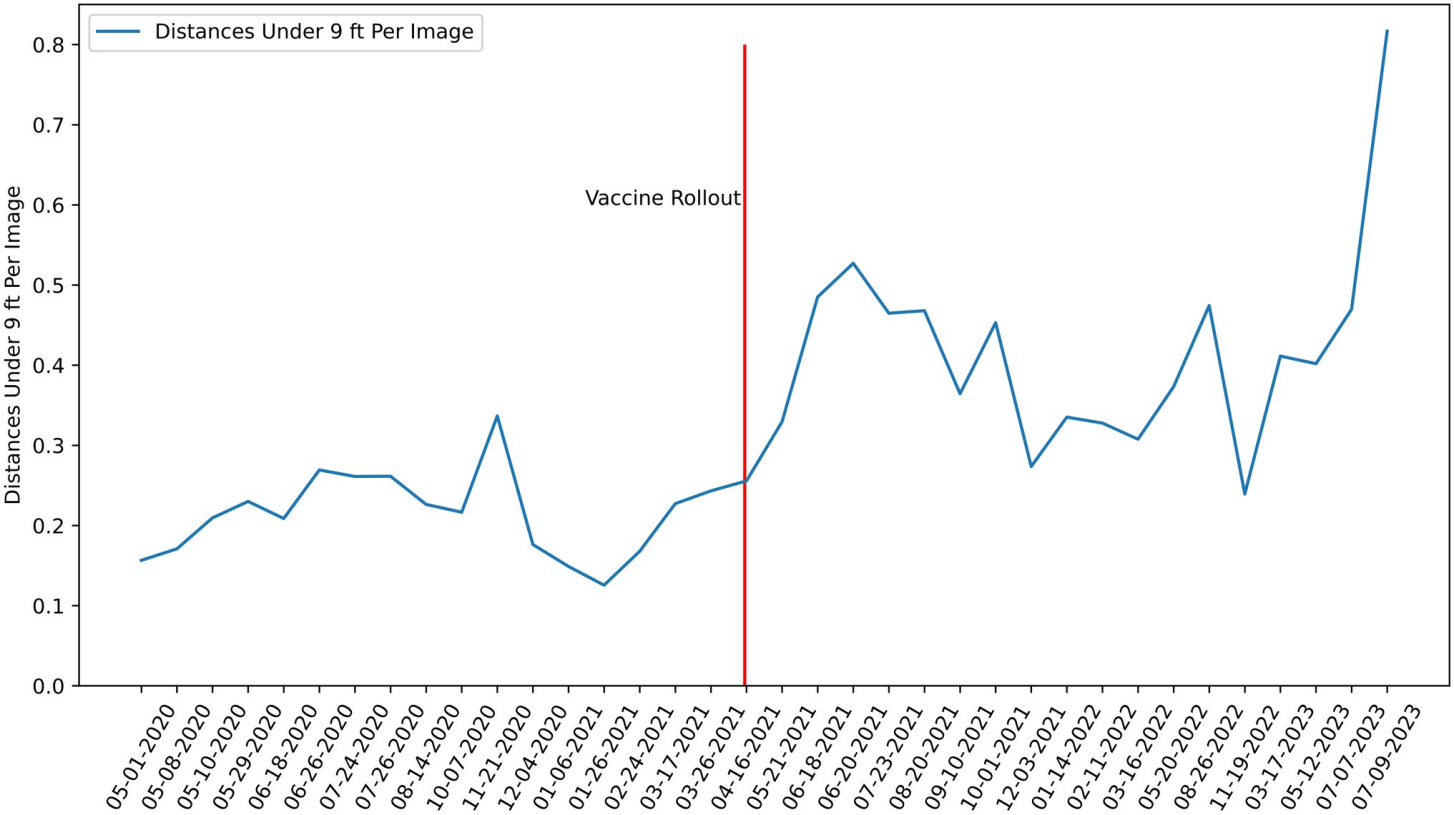
Summary data from the outdoor social distancing analysis. The blue line represents the number of distances under 9 ft (2.74 m) per image. For example, 1.0 means that there is one pair of pedestrians on average per image, whose distance is under 9 ft.

The primary data set utilized in this study is the longitudinal street view image data set from Martell et al. [26]. The data set consists of 37 street-view surveys of the city of Seattle, beginning in May 2020 and ending in July 2023 (typically 2–4 weeks apart, as shown in Fig 2). There are over four million time-stamped, location-tagged images across the 37 surveys. 36 of the 37 data collection surveys were used in this study, as a heavy rain event caused a survey to be stopped early on 10–29-2020. The route design is featured in Errett et al. [28]. As described in Martell et al. [26], we created a data pipeline that took the 360-degree street view images and identified pedestrians in them using the Pedestron algorithm [29]. The output of this pipeline was a set of bounding boxes (each of which is a set of 4 coordinates surrounding a pedestrian, as seen in Fig 1). Additional data features included latitude, longitude, GEOID [30] of the census tract where the image was captured, and various demographic data related to that census tract from the 2019 American Community Survey. Other data features include day of the week, and season of the year, both directly derived from the image timestamp. We consulted with the University of Washington Human Subjects Division, to determine that this study was not considered human subjects research. As such, it did not require Institutional Review Board approval. The data captured in Martell et al. [26] was people in public places, where they cannot expect personal privacy. The image data was published through Mapillary, which automatically obscures faces as an added precaution. For additional ethical reflections, please see the Discussion section.

Summary statistics for each of the predictor variables are seen in Tables 1 and 2. To operationalize this data, we converted the tract-specific data to binary indicators based on Jenk’s natural breaks optimization used during the route design [28]. The day of the week variable was converted to be a binary variable for whether it was the weekend or not. Based on previous analysis [26], we converted the season variable to be a binary for whether it was summer or not.

**Table 1:** Summary statistics for run-specific data.

Number of Runs on Weekdays	31
Number of Runs on Weekends	5
Number of Runs before Vaccine	17
Number of Runs after Vaccine	19
Number of Runs in the Summer	10
Number of Runs in other Seasons	26

**Table 2:** Summary statistics for tract-specific data.

n = 102 Tracts	Minimum	Median	Maximum	Mean
Median Income (2019 Dollars)	$17,188	$96,922	$208,636	$97,259
Percentage of Population that Identifies as White	10.2%	73.45%	90.7%	66.4%

In addition to the data pipeline outputs, this study makes use of publicly available geolocation data on community capital transects from the city of Seattle. These capitals are theorized to be closely tied to community resilience [31] and were used in the design of the data collection survey route. The capitals examined in this study are parks, schools, faith-based organizations, museums, hospitals, medical clinics, and transit stops. Data for these capitals are publicly available through King County GIS Data Hub [32], Washington State Department of Health [33], the City of Seattle [34], and the Association of Religion Data Archives [35]. These locations were chosen to provide coverage over natural, cultural, and built capitals [36], and to provide a view into patterns at public health infrastructure.

We further processed the data by calculating distances between detected bounding boxes. We used the technique of Salazar [27] to obtain estimates of distances between bounding boxes, even in a 2D image. This technique is based on geometric properties and utilizes the Pythagorean Theorem to estimate distances. To validate the algorithm, we applied Salazar’s method to an experimental ground truth dataset. The Root Mean Square Error (RMSE) on this data set was 1.13 ft. The ground truth data set was collected using the same equipment and methods as the data set utilized in this study. Applying this method to our data resulted in almost 5 million distance calculations. Lastly, we subset the distances to look at only those estimated by the algorithm to be under 9 ft (2.74 m) in length. We decided on this subset as a conservative estimate, using the six ft apart guideline provided by the CDC and the 1.13 ft (0.34 m) RMSE of the distance calculation algorithm [27]. With this RMSE, we can be confident that anything measured as 9 ft or greater by the algorithm is at least 6 ft apart in reality.

After we calculated the distances, we paired the distances with various capitals based on geolocation. A distance is assumed to be at a given capital if it is within 200 ft (60.96 m) of the shapefile footprint of that capital. The one exception to this is transit stops, which we only allowed for a 15 ft (4.57 m) distance as pedestrians usually wait very near to, or inside of transit stops. Additionally, these structures have much smaller footprints when compared to the other analyzed capitals. The last step in data preparation was to normalize the distances counts by the number of images captured in a given census tract. While the survey route was the same from run to run, the exact number of images captured at a given location may not be. Normalizing the number of distances per image for each survey run can help alleviate this inconsistency.

### Exploratory data analysis

Before conducting regression analyses on social distancing behaviors, we conducted an exploratory data analysis to better understand trends in the data set. Fig 2 was a key input into our initial modeling decisions. First, we noticed that the number of distances under 9 ft increases sharply in April 2021 and stays high over the rest of the surveys. This corresponds with when the initial COVID-19 vaccine became publicly available for all over the age of 16 in the state of Washington. While it is not strongly visible, we also expected there likely will be some seasonality present in the data, particularly an increase in traffic during the summer months. Lastly, while not easily visible in the figure, we expected there will be a relationship between the day of the week and community mobility. Beyond what we could glean from the graph, there are known inequities in how the COVID-19 pandemic affected different racial groups and people of different income levels [37]. Thus, we wanted to make sure to include all of the above factors as predictors in our models.

### Regression analyses

Based on the initial analysis, we developed the following regression model to identify which factors are statistically significant (*α* = .05):

$$\begin{array}{c} \begin{array}{c} Y=\beta_{0}+\beta_{1}\times I_{vaccine}+\beta_{2}\times C_{season}+\beta_{3}\times I_{weekend}\\ +\beta_{4}\times C_{incomelevel}+\beta_{5}\times I_{demographicindicator}+\epsilon , \end{array} \end{array}$$ (1)

where *Y* is the number of distances under 9 ft per image for each date/census tract combination; *I*_*vaccine*_ is an indicator for if the vaccine was available on that date; *C*_*season*_ is an indicator variable for if it is summer or not; *I*_*weekend*_ is an indicator for if it is the weekend or not; *C*_*incomelevel*_ is an indicator variable for if a given census tract has a median income above $80,820; *I*_*demographicindicator*_ is an indicator variable for if the population is 55.5% white or more. The $80,820 and 55.5% breakpoints were chosen based on Jenk’s natural breaks optimization. *β*_0_ is the baseline number of distances per image on a weekday, not in the summer, with the vaccine unavailable, in a census tract with income below $80,820 and a population less than 55.5% white. *β*_1_ represents the change in the number of distances per image from the vaccine becoming available, and *β*_2_ represents the change for it being the summer. *β*_3_ represents the change from a weekday to the weekend, and *β*_4_ represents the change to the higher income bracket. Lastly, *β*_5_ represents the change from an area that is less than 55.5% white to an area that is more.

We utilized this model on the entire data set, as well as data subsets located at the various community capitals of interest. We thought it possible that different community capitals would experience different trends over time, and different seasonality effects. As an example, traffic around schools is likely to decrease in the summers rather than increase like we expected to see in the full model. We also developed a similar model for the proportion of the distances under 9 ft. While this model does violate the linearity assumption of a linear regression model, the predicted outputs are all well within the 0–1 bounds of a proportion. We employ a simple form of regression model, as our focus is on drawing inferences rather than making predictions. The only other change from the above model is that the regression coefficients are interpreted as changes in predicted proportion, rather than number of distances per image.

In addition to the above regression models, we examined the baseline trends in social distancing patterns at different capital locations. We did this by first calculating the overall proportion of distances under 9 ft for the entire data set and at each community capital. Then we compared the proportions at each capital to the overall proportion using a difference in proportions test. This allowed us to have a baseline understanding of the differences between the community capitals.

## Results

### Distances under 9 ft per image

The regression analysis results for the number of distances under 9 ft per image across the entire data set are displayed in Table 3. Vaccine availability, whether it was the weekend or not, it being summer or not, and proportion of the population that identified as white were all significant, positive predictors. Income level was a significant, negative predictor. Results for individual capitals are summarized in Table 4, with full regression outputs in the S1 File.

**Table 3:** OLS regression results—Distances under 9 ft per image. Please note that as there is some overlap that occurs between images, the coefficients here can only be interpreted relative to each other. Stating that the vaccine effect is 3 times as great as the summer effect in the same direction is useful, but the absolute interpretation of these coefficients’ sizes is not. Full documentation for the Python package used to make this tabular output is available from the developers [38].

**Dep. Variable**:	Distances Under 9 ft Per Image			**R-squared**:			0.036
**Model**:	OLS			**Adj. R-squared**:			0.034
**Method**:	Least Squares			**F-statistic**:			23.53
**No. Observations**:	3171			**Prob (F-statistic)**:			2.69e-23
**Df Residuals**:	3165			**Log-Likelihood**:			-3306.3
**Df Model**:	5						
**Covariance Type**:	nonrobust						
	**coef**	**std err**	**t**	**P> |t|**	**[0.025**	**0.975]**	
**Intercept**	0.1753	0.032	5.405	0.000	0.112	0.239	
**Summer**	0.0540	0.027	1.968	0.049	0.000	0.108	
**Vaccine Available**	0.1686	0.025	6.824	0.000	0.120	0.217	
**Weekend**	0.1000	0.036	2.807	0.005	0.030	0.170	
**Income Above $80,820**	-0.1969	0.029	-6.797	0.000	-0.254	-0.140	
**More than 55.5% White**	0.1925	0.032	5.937	0.000	0.129	0.256	

**Table 4:** OLS regression results—Distances under 9 ft per image—Summary of capitals analysis. The numbers reported are the regression coefficents. Significance level: 0.05*, 0.01**, or <0.001***.

Capital	Intercept	Summer	Vaccine Available	Weekend	Income Above $80,820	More than 55% White
**Transit Stops**	0.0313***	0.0113	0.0262***	0.0045	-0.0261**	0.0077
**Parks**	0.0216	0.0007	0.0478***	0.0248	0.0043	0.0430**
**Schools**	0.0024	-0.0149*	0.0214**	-0.0126	0.0152	0.0093
**Hospitals**	-0.0002	0.0063	-0.0014	-0.0045	0.0048	0.0160
**Medical Clinics**	0.0940***	0.0029	0.0606**	0.0101	-0.1342***	0.0606**
**Faith-Based Organizations**	0.0270**	0.0189*	0.0286***	0.0257**	-0.0276***	0.0188*
**Museums**	-0.0249	0.0253	0.1094***	0.1075**	0.1915***	-0.0331

There were a few notable results from the regression analyses at community capitals. One immediately noticeable result from Table 4 was that vaccine availability was significant in all models, except at hospitals. In fact, hospitals did not have any significant predictors at all. Museums were the only capital to have a significant, positive income effect. Schools had a significant, negative effect from it being summer, and faith-based organizations had comparably sized vaccine and weekend effects. Lastly, when compared to the other capitals, museums had substantially larger coefficients for vaccine availability, the weekend effect, and the income effect.

### Proportion of distances under 9 ft

To obtain the proportion of distances less than 9 ft, we simply calculated the ratio between the number of distances under 9 ft, and the total number of distances. For the regression analysis, we subset the data at the survey and census tract levels first, then calculated the proportions. The proportion of distances less than 9 ft across the entire data set is 0.264. We then performed the same procedure for only distances at each specific capital and compared them to the total using a difference in proportions test. The results are displayed in Table 5.

**Table 5:** Difference-in-proportions test results for the proportion of distances under 9 ft *at each capital*, when compared to 0.264, the proportion of distances under 9 ft across the *entire* data set.

Capital	Proportion	p-value
**Transit Stops**	0.271	<.00001
**Parks**	0.256	<.00001
**Schools**	0.228	<.00001
**Hospitals**	0.279	<.00001
**Medical Clinics**	0.264	0.90242
**Faith-Based Organizations**	0.285	<.00001
**Museums**	0.261	.014509

The regression analysis results for the proportion of distances under 9 ft across the entire data set are displayed in Table 6. Vaccine availability, whether it was the weekend or not, and income level were all significant, positive predictors. It being summer was a significant, negative predictor. Results for individual capitals are summarized in Table 7, with full regression outputs in the S1 File.

**Table 6:** OLS regression results—Proportion of distances under 9 ft. The coefficients here can be directly interpreted as the relative change in the proportion of distances under 9 ft when a given predictor changes from 0 to 1.

**Dep. Variable**:	Proportion of Distances Under 9 ft			**R-squared**:			0.047
**Model**:	OLS			**Adj. R-squared**:			0.046
**Method**:	Least Squares			**F-statistic**:			31.24
**No. Observations**:	3171			**Prob (F-statistic)**:			3.81e-31
**Df Residuals**:	3165			**Log-Likelihood**:			848.19
**Df Model**:	5						
**Covariance Type**:	nonrobust						
	**coef**	**std err**	**t**	**P> |t|**	**[0.025**	**0.975]**	
**Intercept**	0.3168	0.009	36.197	0.000	0.300	0.334	
**Summer**	-0.0287	0.007	-3.882	0.000	-0.043	-0.014	
**Vaccine Available**	0.0388	0.007	5.816	0.000	0.026	0.052	
**Weekend**	0.0258	0.010	2.684	0.007	0.007	0.045	
**Income Above $80,820**	0.0786	0.008	10.057	0.000	0.063	0.094	
**More than 55.5% White**	-0.0120	0.009	-1.368	0.171	-0.029	0.005	

**Table 7:** OLS regression results—Proportion of distances under 9 ft—Summary of capitals analysis. The numbers reported are the regression coefficients. Significance level: 0.05*, 0.01**, or <0.001***.

Capital	Intercept	Summer	Vaccine Available	Weekend	Income Above $80,820	More than 55% White
**Transit Stops**	0.2890***	-0.0355	0.0495**	0.0291	0.0449*	0.0199
**Parks**	0.3452***	-0.0105	0.0280*	0.0412*	0.0327*	-0.0309
**Schools**	0.4857***	-0.0312	-0.0144	0.0873*	0.0467	-0.0329
**Hospitals**	0.3317***	-0.0435	0.0713*	0.1386**	0.0313	-0.927
**Medical Clinics**	0.2834***	-0.0358	0.0202	0.0636*	0.0343	0.0202
**Faith-Based Organizations**	0.3258***	-0.0171	0.0311*	0.0290	0.0436**	-0.0006
**Museums**	0.3155***	-0.0115	0.0528	0.0199	-0.0187	0.0197

A notable trend from the community capitals regressions is how the weekend effect being significant in 4 different models, with a larger regression coefficient than the vaccine effect. Demographic variables played less of an effect when compared to the regression for number of distances under 9 ft per image. The income effect was only significant in 3 models, and the proportion of the population identifying as white was not significant in any of the models. The vaccine effect was only significant in 4 models, compared to 6 in the number of distances per image models. In all cases, the effect was still positive when significant.

## Discussion

### Implications

**Fig 3:**
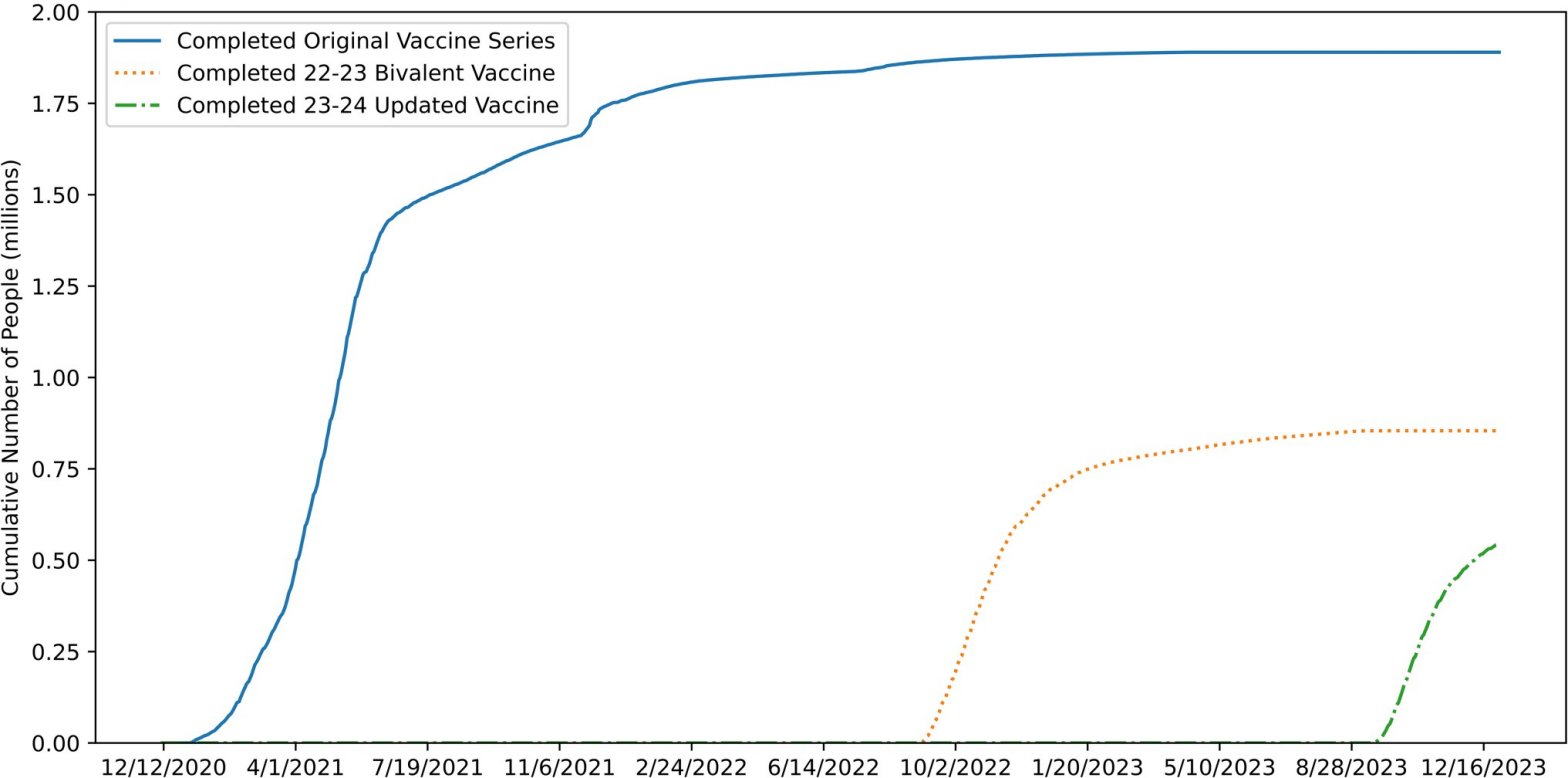
Cumulative vaccination totals over time in King County, Washington, where Seattle is the largest city, show diminishing willingness to get vaccines. Data collected from the King County Department of Public Health [40].

Our results represent important knowledge for researchers and policymakers. First, our approach represents a novel way of understanding outdoor social distancing behaviors. Past research includes looking at worldwide trends over a shorter period of time [23], smaller areas of a city over shorter periods of time (months) [13, 24], and frequently relies on CCTV data being available from public sources [13, 23, 25], or manual data collection and labeling [39]. In comparison, our study analyzes outdoor social distancing over a period of three years across a large portion of a metropolitan area. Our regression analysis shows that across the entire city of Seattle, vaccine availability correlated with increased pedestrian activity. Across community capitals, there was an increase in pedestrians being within 9 ft of each other following the public availability of the vaccine in April 2021. Additionally, the overall proportion of distances that were 9 ft or less also increased. This suggests that, even outdoors, people were more likely to accept the risks of being near one another after the vaccine became available. These results also confirm findings from other studies that show that people were more likely to be willing to take risky behaviors such as visiting crowded places and being near others after being vaccinated [18, 19]. This is not the only additional risk people in the Seattle area are taking as the pandemic has continued, people have been more willing to embrace infection risk by not getting updated vaccines [40]. Fig 3 shows a stark contrast in the number of people who got the original COVID-19 vaccine series compared to the more recent bivalent boosters. In general, as time has gone on it appears that people are less concerned about COVID-19, possibly due to pandemic fatigue [41, 42].

There are known inequities in how COVID-19 affected different communities, including higher mortality rates and infection rates for historically disadvantaged groups [14]. Across income groups, driving factors include the inability to work from home, challenges securing housing at all, and challenges securing quality healthcare for lower income groups [43]. Our analysis shows that lower income areas had more pedestrians traveling within 9 ft of each other, and more pedestrian traffic overall, leading to greater risk of exposure. In contrast, higher income areas did have a higher proportion of distances less than 9 ft.

**Fig 4:**
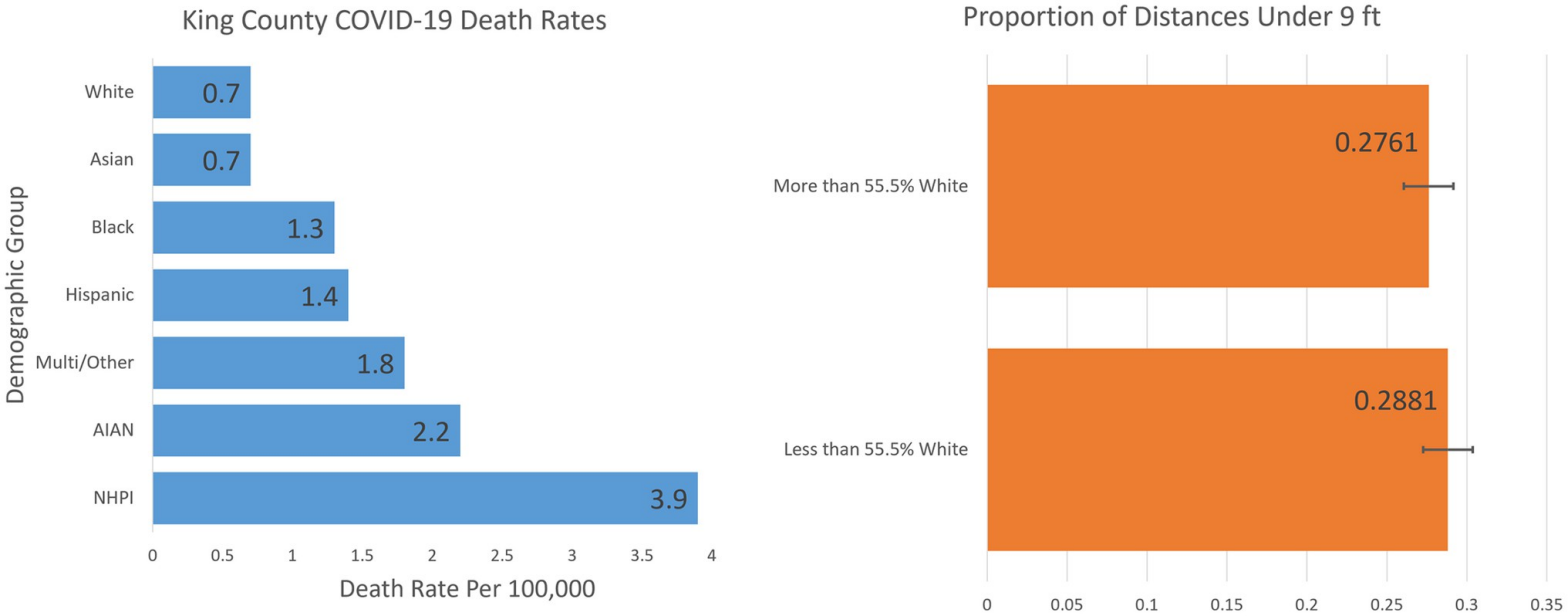
The left side graph shows the death rates per 100,000 citizens for different racial groups in King County, WA [47]. (NHPI, Native Hawaiian or Pacific Islander; AIAN, American Indian or Alaska Native). The right side graph shows the expected proportion of distances under 9 ft per image across our entire data set during the summer, in a lower-income census tract, on a weekday, before the vaccine became publicly available, based on our regression results. It is shown here that the more white areas did not have a statistically significant difference from the less white areas.

Racial demographics were not shown to significantly predict the proportion of distances under 9 ft. In contrast, census tracts that had a larger population identifying as white had more distances under 9 ft per image across the entire data set, and at parks, medical clinics, and faith-based organizations. This is a somewhat surprising result, as is the higher proportion of distances under 9 ft in higher income areas. The known racial disparity in COVID-19 health outcomes in King County (see Fig 4) and racial and socioeconomic disparities in the US as a whole [37], would lead us to expect the opposite. However, it is also known that communities of color are more likely to wear a mask, and that the drivers behind higher infection rates and death are systemic inequities in wealth, income, underlying health conditions, social capital, and lower quality health care [44–46]. In this light, it is less surprising that rates of social distancing adherence across areas with different racial demographics and income levels did not directly correlate with the divergent health outcomes in those groups.

Lastly, the findings from this study show some expected results with regard to weekend effects and seasonality. Across the data set, weekend surveys had higher numbers of distances under 9 ft, and the proportion of distances under 9 ft was also higher. These results held true at many, but not all, the capitals of interest. In the summer, the effect was mixed, with the total number of distances under 9 ft increasing across the entire data set, even though the proportion of distances under 9 ft decreased. This suggests that increased foot traffic and decreased social distancing adherence is not a given relationship when outdoors. In general, it is easier to maintain physical distance when outdoors when compared to being indoors, even if foot traffic is somewhat higher.

When comparing the social distancing results at different community capitals, two results stand out. First is the substantially lower proportion of distances less than 9 ft at schools. Schools have authority to enforce strict social distancing rules over their students. While indoors this could prove problematic for a variety of reasons [48], but outdoors, in our study area, these rules are much easier to enforce. Schools also experienced expected results in the regression analyses, with summer being a driver for a decrease in distances under 9 ft. In contrast, faith-based organizations were the location with the highest proportion of defections within 9 ft of each other. The relationship between religious groups and COVID-19 related restrictions in the U.S. can be described as tenuous at best [49], with frequent legal battles over restrictions. Additionally, there is a known link between highly religious Americans (particularly evangelicals) and less concern and support for policy recommendations from public health officials [50]. In this light, it is somewhat expected that social distancing recommendations would be less strictly followed at faith-based organizations compared to other community capitals. However, the regression results at faith-based organizations showed that both the number of distances under 9 ft and the proportion under 9 ft increased after the vaccine became available. Thus, there still was some level of risk avoiding behavior at faith-based organizations prior to vaccine availability. Faith-based organizations experienced an expected result of an increase in distances on weekends.

There were other notable results related to different community capitals from the regression analyses. First, hospitals did not have any significant predictors for the number of distances under 9 ft. Hospital traffic is largely driven by demand rather than other factors, which would lead to none of the seasonality effects being significant. In terms of socioeconomic predictors, hospitals are not limited to serving people who live in the immediate area, so the demographics of the census tract the institution is located would not be expected to impact foot traffic. The COVID-19 infection rate may be a better predictor for foot traffic at hospitals, although more research is needed to confirm this. In contrast, medical clinics had a vaccine effect, income effect, and racial demographic effect that were similar to the overall data set. This is likely due to the outdoor nature of our data. Medical clinics are much more common and cover more of the city than hospitals do, leading them to trend more with the overall data set. This is another capital where more research could be done to better understand social distancing behaviors. Lastly, the large income effect at museums is likely due to the fact that the majority of museums in Seattle are located in wealthy areas, leading to a natural bias.

Another important item to address are the ethical concerns of this type of research. The data collected for this study was naturalistic observation in public places, and thus not considered human subjects research. This is in line with the definition put forward by the American Sociological Association’s code of ethics [51]. However, this type of large-scale image data collection still raises many privacy concerns [25]. While expected levels of privacy, and privacy regulations, differ across cultures, privacy is valued globally [52]. Thus, while the methods outlined in this study are considered acceptable in the United States, they may not be elsewhere.

Similarly, there are significant differences in what is considered acceptable across disciplines [25]. There is some tension between reproducibility in computer vision research and privacy concerns, with reproducibility usually winning out. The practice of collecting and annotating large amounts of video data in public places, without consent of those recorded, is commonplace in this field. These data sets are shared amongst researchers, frequently without anonymization, to verify and improve upon each other’s work. This is in contrast with social science research, where this practice would be met with harsh scrutiny. Without suggesting that one practice is better than the other, this example does illustrate the gap that currently exists between fields for this type of data collection. As interdisciplinary research becomes more common, these contrasting views are more likely to come into conflict.

### Limitations

One challenge with utilizing computer vision is that the data product created, in this case the number of estimated distances, cannot be interpreted as the actual number of distances at a given location. There is overlap in the image data in Martell et al. [26]. Pedestrians that appear in the foreground of one image may end up in the background of another. However, our results in this study are enough to demonstrate that while the raw number of distances may not be perfect, the relative change over time is meaningful. Additionally, this type of data collection only captures people near drive-able roads. As some locations of interest such as hospitals and parks may have large footprints away from roads, some data on pedestrian traffic near these areas will be lost that would potentially be captured by other methods such as using cell phone data. Our methods serve as a complement to data of this type, not a replacement.

The distance estimation algorithm is also not without problems [27]. The authors utilized an experiment to determine the optimal values for the algorithm’s parameters through a grid search. In their tests, the ground truth pedestrians stood no more than 12 ft apart. The RMSE of 1.13 ft mentioned earlier in the paper is from those conditions. However, in our data set, pedestrians are frequently further apart than 12 ft, and further away from the vehicle than 20 ft. Thus, under those conditions, the algorithm’s validity decreases, sometimes to the point where it overestimates distances by multiple orders of magnitude. However, given the nature of our study, this overestimation is acceptable. As we only care about pedestrians 9 ft apart or closer, we are within the bounds of what the algorithm was trained on. When the overestimation occurs, the estimated distance obtains the same classification it would have otherwise, 9 ft or greater.

Another challenge with this method is the inability to differentiate between people from the same social bubbles, and strangers. During the height of the pandemic, households would form ‘bubbles’ of small non-overlapping groups that would be able to come into contact with each other while still maintaining social distancing benefits [53]. While our methods cannot account for these social bubbles, Danon et al. [53] shows that there are still substantial risks of transmission when this tactic is employed. Given this insight, we argue that this limitation does not substantially harm our findings.

Lastly, while the survey route was designed to provide a good representation of the city of Seattle, it is not without its flaws [28]. There are some key locations in the city that were missed, such as “The Ave” in the University District, that would have been valuable areas to study. Additionally, the route completely omitted West Seattle due to driving time constraints. These omissions do not directly harm the validity of the results in this paper, but they do somewhat limit their scope.

### Extensions

This methodology has the potential to be applied to future pandemic events to understand the impacts on social distancing behaviors and community mobility as a whole. If possible, conducting an occasional baseline survey would allow for pandemic-era data to be compared to pre-pandemic levels, something that was not done for this study. Additionally, this type of analysis can be easily extended to other image data sets, whether it be indoors or outdoors, to understand changes in social distancing behaviors over time at key locations.

There are also still more insights that can be gleaned from this data set. For example, some capitals of interest that were not included in this study, such as cycling infrastructure [54, 55], could be topics of further study. Additionally, large-scale longitudinal street view imagery has a host of applications outside public health such as studying the built environment, and urban analytics [56, 57]. Lastly, with a larger data set, more predictor variables could be analyzed, such as more specific racial demographics than just white vs nonwhite, English proficiency, age and including interaction terms between predictors.

Finally, improvements in generalizable pedestrian detection algorithms or distance estimation in 2-dimensional images would allow for a higher accuracy in model outputs. Improvements in this area could allow for real-world interpretable outputs if pedestrian counts were more accurate. It would also allow for analysis of pedestrians that are further apart from each other, and more confidence in the model outputs for these cases. Similarly, it would become possible to study social distancing patterns for extremely short distances (e.g. less than 3ft) which is currently not possible given the RMSE of the distance estimation algorithm we used. Lastly, algorithms that detect characteristics such as mask-wearing or gender [22] could be used for an individual-level analysis, as opposed to the group-level analysis done here. In a similar vein, street crowding is known to impact pedestrian behavior, as people who are put in situations where distancing is difficult are more likely to violate recommendations [58, 59]. An individual-level variable for crowding would allow our model to provide additional insights.

### Conclusions

This study represents a first of its kind effort to track outdoor social distancing behaviors. We used a longitudinal street-view image survey, computer vision, and distance estimation techniques to generate over four million estimated distances across a 3-year survey period. We show that vaccine availability was a key driver in outdoor social distancing in the city of Seattle, with an increase in the number of distances under 9 ft after the vaccine became publicly available. Our results also highlight some of the systemic inequities that exist within the city that match broader trends in the US. This included that lower income areas experienced higher levels of pedestrians in proximity to each other, and that whiter areas had higher numbers of pedestrians in proximity to each other, in spite of white people still having better COVID-19 related health outcomes. Lastly, we were able to quantify the differences between community capitals with regard to social distancing behaviors. We observed that faith-based organizations had the lowest levels of social distancing adherence, while schools had the highest adherence levels.

## Supporting information

S1 File Supplementary information for outdoor social distancing behavior changed during a pandemic. Additional sample images and full regression output for capitals analysis.
(PDF)

S1 Dataset Dataset used to obtain regression results across the entirety of Seattle. (CSV)

S2 Dataset Dataset used to obtain regression results at faith-based organizations. (CSV)

S3 Dataset Dataset used to obtain regression results at hospitals. (CSV)

S4 Dataset Dataset used to obtain regression results at medical clinics. (CSV)

S5 Dataset Dataset used to obtain regression results at museums. (CSV)

S6 Dataset Dataset used to obtain regression results at parks. (CSV)

S7 Dataset Dataset used to obtain regression results at schools. (CSV)

S8 Dataset Dataset used to obtain regression results at transit stops. (CSV)
