# Supplementary material for: Outdoor social distancing behaviors changed during a pandemic: A longitudinal analysis using street view imagery
Source: PLoS One. 2024 Dec 5;19(12):e0315132. doi: 10.1371/journal.pone.0315132 (PMC11620612; doi:10.1371/journal.pone.0315132)
Supplement: S1 File — Additional sample images and full regression output for capitals analysis. (PDF) [file pone.0315132.s001.pdf]

Supplementary Information for
Outdoor Social Distancing Behaviors Changed During a Pandemic

1 Additional Sample Image Outputs

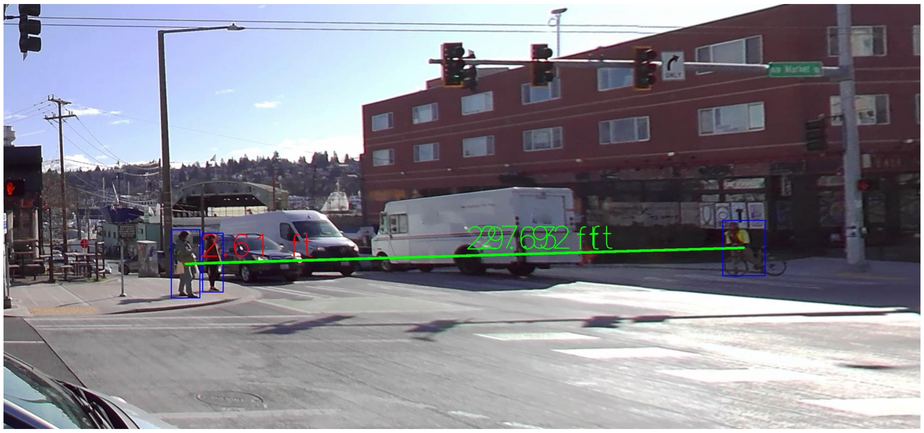

Figure S1: Sample output of the pedestrian detection and physical distancing algorithm. This image contains two pedestrians near each other on one side of the street, about 30 feet away from a pedestrian across the street.

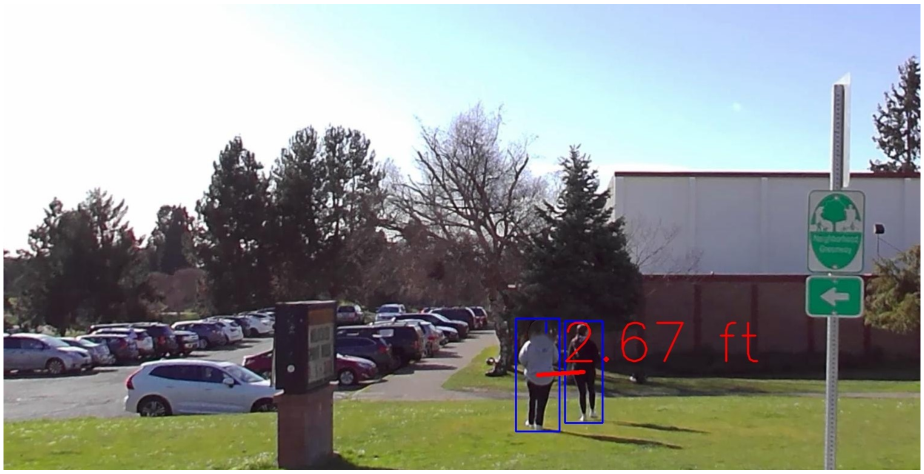

Figure S2: Sample output of the pedestrian detection and physical distancing algorithm. This image contains two pedestrians walking nearby each other with their backs to the camera.

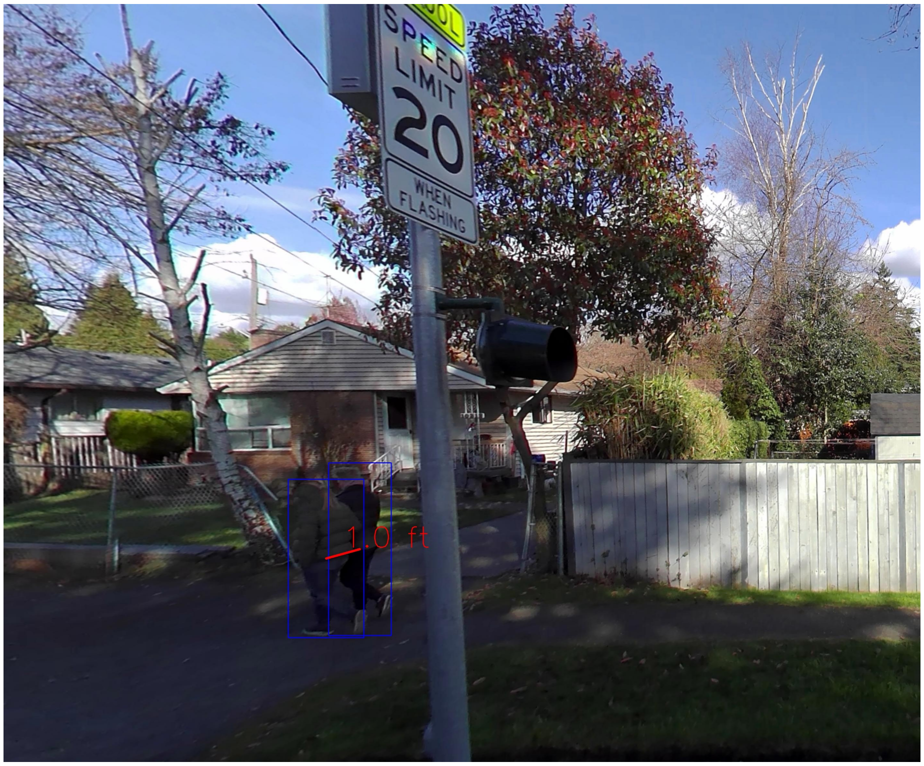

Figure S3: Sample output of the pedestrian detection and physical distancing algorithm. This image contains two pedestrian walking nearby each other walking parallel with the vehicle.

2 Capitals Regression Results - Number of Distances Under 9 ft per Image

**Dep. Variable:**	Short_per_Image	**R-squared:**	0.021
**Model:**	OLS	**Adj. R-squared:**	0.017
**Method:**	Least Squares	**F-statistic:**	5.661
**No. Observations:**	1348	**Prob (F-statistic):**	3.57e-05
**Df Residuals:**	1342	**Log-Likelihood:**	858.81
**Df Model:**	5		
**Covariance Type:**	nonrobust		

	**coef**	**std err**	**t**	**P>|t|**	**[0.025**	**0.975]**
**Intercept**	0.0313	0.009	3.677	0.000	0.015	0.048
**Summer**	0.0113	0.008	1.473	0.141	-0.004	0.026
**Vaccine Available**	0.0262	0.007	3.692	0.000	0.012	0.040
**Weekend**	0.0045	0.011	0.429	0.668	-0.016	0.025
**Income Above $80,820**	-0.0261	0.008	-3.248	0.001	-0.042	-0.010
**More than 55.5% White**	0.0077	0.009	0.860	0.390	-0.010	0.025

Table S1: OLS Regression Results - Transit Stops - Number of Distances Under 9 ft

**Dep. Variable:**	Short_per_Image	**R-squared:**	0.031
**Model:**	OLS	**Adj. R-squared:**	0.027
**Method:**	Least Squares	**F-statistic:**	9.634
**No. Observations:**	1532	**Prob (F-statistic):**	4.55e-09
**Df Residuals:**	1526	**Log-Likelihood:**	953.40
**Df Model:**	5		
**Covariance Type:**	nonrobust		

	**coef**	**std err**	**t**	**P>|t|**	**[0.025**	**0.975]**
**Intercept**	0.0270	0.008	3.350	0.001	0.011	0.043
**Summer**	0.0189	0.007	2.533	0.011	0.004	0.034
**Vaccine Available**	0.0286	0.007	4.234	0.000	0.015	0.042
**Weekend**	0.0257	0.010	2.625	0.009	0.007	0.045
**Income Above $80,820**	-0.0276	0.007	-3.730	0.000	-0.042	-0.013
**More than 55.5% White**	0.0188	0.008	2.399	0.017	0.003	0.034

Table S2: OLS Regression Results - Faith-Based Organizations - Number of Distances Under 9 ft

**Dep. Variable:**	Short_per_Image	**R-squared:**	0.028
**Model:**	OLS	**Adj. R-squared:**	0.010
**Method:**	Least Squares	**F-statistic:**	1.545
**No. Observations:**	275	**Prob (F-statistic):**	0.176
**Df Residuals:**	269	**Log-Likelihood:**	562.77
**Df Model:**	5		
**Covariance Type:**	nonrobust		

	**coef**	**std err**	**t**	**P> |t|**	**[0.025**	**0.975]**
**Intercept**	-0.0002	0.009	-0.019	0.985	-0.019	0.019
**Summer**	0.0063	0.004	1.492	0.137	-0.002	0.015
**Vaccine Available**	-0.0014	0.004	-0.360	0.719	-0.009	0.006
**Weekend**	-0.0045	0.006	-0.792	0.429	-0.016	0.007
**Income Above $80,820**	0.0048	0.004	1.223	0.223	-0.003	0.013
**More than 55.5% White**	0.0160	0.010	1.661	0.098	-0.003	0.035

Table S3: OLS Regression Results - Hospitals - Number of Distances Under 9 ft

**Dep. Variable:**	Short_per_Image	**R-squared:**	0.072
**Model:**	OLS	**Adj. R-squared:**	0.066
**Method:**	Least Squares	**F-statistic:**	12.42
**No. Observations:**	809	**Prob (F-statistic):**	1.25e-11
**Df Residuals:**	803	**Log-Likelihood:**	-19.400
**Df Model:**	5		
**Covariance Type:**	nonrobust		

	**coef**	**std err**	**t**	**P> |t|**	**[0.025**	**0.975]**
**Intercept**	0.0940	0.020	4.612	0.000	0.054	0.134
**Summer**	0.0029	0.020	0.149	0.882	-0.035	0.041
**Vaccine Available**	0.0606	0.018	3.431	0.001	0.026	0.095
**Weekend**	0.0101	0.025	0.395	0.693	-0.040	0.060
**Income Above $80,820**	-0.1342	0.019	-7.050	0.000	-0.172	-0.097
**More than 55.5% White**	0.0606	0.021	2.845	0.005	0.019	0.102

Table S4: OLS Regression Results - Medical Clinics - Number of Distances Under 9 ft

**Dep. Variable:**	Short_per_Image	**R-squared:**	0.190
**Model:**	OLS	**Adj. R-squared:**	0.176
**Method:**	Least Squares	**F-statistic:**	13.52
**No. Observations:**	294	**Prob (F-statistic):**	7.46e-12
**Df Residuals:**	288	**Log-Likelihood:**	32.332
**Df Model:**	5		
**Covariance Type:**	nonrobust		

	**coef**	**std err**	**t**	**P> |t|**	**[0.025**	**0.975]**
**Intercept**	-0.0249	0.025	-0.983	0.326	-0.075	0.025
**Summer**	0.0253	0.029	0.880	0.380	-0.031	0.082
**Vaccine Available**	0.1094	0.026	4.231	0.000	0.058	0.160
**Weekend**	0.1075	0.038	2.817	0.005	0.032	0.183
**Income Above $80,820**	0.1915	0.035	5.448	0.000	0.122	0.261
**More than 55.5% White**	-0.0331	0.034	-0.967	0.334	-0.100	0.034

Table S5: OLS Regression Results - Museums - Number of Distances Under 9 ft

**Dep. Variable:**	Short_per_Image	**R-squared:**	0.026
**Model:**	OLS	**Adj. R-squared:**	0.023
**Method:**	Least Squares	**F-statistic:**	9.512
**No. Observations:**	1783	**Prob (F-statistic):**	5.75e-09
**Df Residuals:**	1777	**Log-Likelihood:**	434.16
**Df Model:**	5		
**Covariance Type:**	nonrobust		

	**coef**	**std err**	**t**	**P> |t|**	**[0.025**	**0.975]**
**Intercept**	0.0216	0.011	1.915	0.056	-0.001	0.044
**Summer**	0.0007	0.010	0.065	0.948	-0.019	0.021
**Vaccine Available**	0.0478	0.009	5.238	0.000	0.030	0.066
**Weekend**	0.0248	0.013	1.873	0.061	-0.001	0.051
**Income Above $80,820**	0.0043	0.011	0.376	0.707	-0.018	0.026
**More than 55.5% White**	0.0430	0.013	3.415	0.001	0.018	0.068

Table S6: OLS Regression Results - Parks - Number of Distances Under 9 ft

**Dep. Variable:**	Short_per_Image	**R-squared:**	0.031
**Model:**	OLS	**Adj. R-squared:**	0.026
**Method:**	Least Squares	**F-statistic:**	5.611
**No. Observations:**	871	**Prob (F-statistic):**	4.24e-05
**Df Residuals:**	865	**Log-Likelihood:**	855.59
**Df Model:**	5		
**Covariance Type:**	nonrobust		

	**coef**	**std err**	**t**	**P> |t|**	**[0.025**	**0.975]**
**Intercept**	0.0024	0.009	0.281	0.779	-0.014	0.019
**Summer**	-0.0149	0.007	-2.200	0.028	-0.028	-0.002
**Vaccine Available**	0.0214	0.006	3.428	0.001	0.009	0.034
**Weekend**	-0.0126	0.009	-1.343	0.180	-0.031	0.006
**Income Above $80,820**	0.0152	0.010	1.450	0.148	-0.005	0.036
**More than 55.5% White**	0.0093	0.010	0.923	0.356	-0.010	0.029

Table S7: OLS Regression Results - Schools - Number of Distances Under 9 ft

3 Capitals Regression Results - Proportion of Distances Under 9 ft

**Dep. Variable:**	Prop_Short	**R-squared:**	0.014
**Model:**	OLS	**Adj. R-squared:**	0.010
**Method:**	Least Squares	**F-statistic:**	3.682
**No. Observations:**	1348	**Prob (F-statistic):**	0.00259
**Df Residuals:**	1342	**Log-Likelihood:**	-427.14
**Df Model:**	5		
**Covariance Type:**	nonrobust		

	**coef**	**std err**	**t**	**P> |t|**	**[0.025**	**0.975]**
**Intercept**	0.2890	0.022	13.081	0.000	0.246	0.332
**Summer**	-0.0355	0.020	-1.776	0.076	-0.075	0.004
**Vaccine Available**	0.0495	0.018	2.688	0.007	0.013	0.086
**Weekend**	0.0291	0.027	1.064	0.288	-0.025	0.083
**Income Above $80,820**	0.0449	0.021	2.150	0.032	0.004	0.086
**More than 55.5% White**	0.0199	0.023	0.860	0.390	-0.025	0.065

Table S8: OLS Regression Results - Transit Stops - Proportion of Distances Under 9 ft

**Dep. Variable:**	Prop_Short	**R-squared:**	0.009
**Model:**	OLS	**Adj. R-squared:**	0.006
**Method:**	Least Squares	**F-statistic:**	2.854
**No. Observations:**	1532	**Prob (F-statistic):**	0.0143
**Df Residuals:**	1526	**Log-Likelihood:**	-235.43
**Df Model:**	5		
**Covariance Type:**	nonrobust		

	**coef**	**std err**	**t**	**P> |t|**	**[0.025**	**0.975]**
**Intercept**	0.3258	0.018	18.615	0.000	0.291	0.360
**Summer**	-0.0171	0.016	-1.057	0.291	-0.049	0.015
**Vaccine Available**	0.0311	0.015	2.117	0.034	0.002	0.060
**Weekend**	0.0290	0.021	1.359	0.174	-0.013	0.071
**Income Above $80,820**	0.0436	0.016	2.708	0.007	0.012	0.075
**More than 55.5% White**	-0.0006	0.017	-0.034	0.973	-0.034	0.033

Table S9: OLS Regression Results - Faith-Based Organizations - Proportion of Distances Under 9 ft

**Dep. Variable:**	Prop_Short	**R-squared:**	0.052
**Model:**	OLS	**Adj. R-squared:**	0.034
**Method:**	Least Squares	**F-statistic:**	2.928
**No. Observations:**	275	**Prob (F-statistic):**	0.0136
**Df Residuals:**	269	**Log-Likelihood:**	-21.084
**Df Model:**	5		
**Covariance Type:**	nonrobust		

	**coef**	**std err**	**t**	**P> |t|**	**[0.025**	**0.975]**
**Intercept**	0.3317	0.079	4.183	0.000	0.176	0.488
**Summer**	-0.0435	0.035	-1.228	0.221	-0.113	0.026
**Vaccine Available**	0.0713	0.032	2.214	0.028	0.008	0.135
**Weekend**	0.1386	0.048	2.915	0.004	0.045	0.232
**Income Above $80,820**	0.0313	0.033	0.948	0.344	-0.034	0.096
**More than 55.5% White**	-0.0927	0.080	-1.153	0.250	-0.251	0.066

Table S10: OLS Regression Results - Hospitals - Proportion of Distances Under 9 ft

**Dep. Variable:**	Prop_Short	**R-squared:**	0.017
**Model:**	OLS	**Adj. R-squared:**	0.011
**Method:**	Least Squares	**F-statistic:**	2.811
**No. Observations:**	809	**Prob (F-statistic):**	0.0159
**Df Residuals:**	803	**Log-Likelihood:**	-76.898
**Df Model:**	5		
**Covariance Type:**	nonrobust		

	**coef**	**std err**	**t**	**P> |t|**	**[0.025**	**0.975]**
**Intercept**	0.2834	0.022	12.953	0.000	0.240	0.326
**Summer**	-0.0358	0.021	-1.709	0.088	-0.077	0.005
**Vaccine Available**	0.0202	0.019	1.064	0.288	-0.017	0.057
**Weekend**	0.0636	0.027	2.326	0.020	0.010	0.117
**Income Above $80,820**	0.0343	0.020	1.678	0.094	-0.006	0.074
**More than 55.5% White**	0.0202	0.023	0.883	0.377	-0.025	0.065

Table S11: OLS Regression Results - Medical Clinics - Proportion of Distances Under 9 ft

**Dep. Variable:**	Prop_Short	**R-squared:**	0.012
**Model:**	OLS	**Adj. R-squared:**	-0.005
**Method:**	Least Squares	**F-statistic:**	0.6991
**No. Observations:**	294	**Prob (F-statistic):**	0.625
**Df Residuals:**	288	**Log-Likelihood:**	-10.138
**Df Model:**	5		
**Covariance Type:**	nonrobust		

	**coef**	**std err**	**t**	**P> |t|**	**[0.025**	**0.975]**
**Intercept**	0.3155	0.029	10.770	0.000	0.258	0.373
**Summer**	-0.0115	0.033	-0.347	0.729	-0.077	0.054
**Vaccine Available**	0.0528	0.030	1.769	0.078	-0.006	0.112
**Weekend**	0.0199	0.044	0.452	0.651	-0.067	0.107
**Income Above $80,820**	-0.0187	0.041	-0.461	0.645	-0.099	0.061
**More than 55.5% White**	0.0197	0.040	0.498	0.619	-0.058	0.097

Table S12: OLS Regression Results - Museums - Proportion of Distances Under 9 ft

**Dep. Variable:**	Prop_Short	**R-squared:**	0.008
**Model:**	OLS	**Adj. R-squared:**	0.005
**Method:**	Least Squares	**F-statistic:**	2.866
**No. Observations:**	1783	**Prob (F-statistic):**	0.0139
**Df Residuals:**	1777	**Log-Likelihood:**	-123.56
**Df Model:**	5		
**Covariance Type:**	nonrobust		

	**coef**	**std err**	**t**	**P> |t|**	**[0.025**	**0.975]**
**Intercept**	0.3452	0.015	22.379	0.000	0.315	0.376
**Summer**	-0.0105	0.014	-0.757	0.449	-0.038	0.017
**Vaccine Available**	0.0280	0.012	2.248	0.025	0.004	0.052
**Weekend**	0.0412	0.018	2.273	0.023	0.006	0.077
**Income Above $80,820**	0.0327	0.015	2.112	0.035	0.002	0.063
**More than 55.5% White**	-0.0309	0.017	-1.794	0.073	-0.065	0.003

Table S13: OLS Regression Results - Parks - Proportion of Distances Under 9 ft

**Dep. Variable:**	Prop_Short	**R-squared:**	0.010
**Model:**	OLS	**Adj. R-squared:**	0.005
**Method:**	Least Squares	**F-statistic:**	1.789
**No. Observations:**	871	**Prob (F-statistic):**	0.113
**Df Residuals:**	865	**Log-Likelihood:**	-326.10
**Df Model:**	5		
**Covariance Type:**	nonrobust		

	**coef**	**std err**	**t**	**P>|t|**	**[0.025**	**0.975]**
**Intercept**	0.4857	0.033	14.536	0.000	0.420	0.551
**Summer**	-0.0312	0.026	-1.185	0.236	-0.083	0.020
**Vaccine Available**	-0.0144	0.024	-0.594	0.552	-0.062	0.033
**Weekend**	0.0873	0.036	2.394	0.017	0.016	0.159
**Income Above $80,820**	0.0467	0.041	1.148	0.251	-0.033	0.127
**More than 55.5% White**	-0.0329	0.039	-0.844	0.399	-0.109	0.044

Table S14: OLS Regression Results - Schools - Proportion of Distances Under 9 ft

4 Regression Results - All Distances Per Image

**Dep. Variable:**	Distances_per_Image	**R-squared:**	0.027
**Model:**	OLS	**Adj. R-squared:**	0.025
**Method:**	Least Squares	**F-statistic:**	17.39
**No. Observations:**	3171	**Prob (F-statistic):**	5.01e-17
**Df Residuals:**	3162	**Log-Likelihood:**	-7893.7
**Df Model:**	5		
**Covariance Type:**	nonrobust		

	**coef**	**std err**	**t**	**P> |t|**	**[0.025**	**0.975]**
**Intercept**	0.6767	0.138	4.910	0.000	0.406	0.947
**Summer**	0.2963	0.117	2.541	0.011	0.068	0.525
**Vaccine Available**	0.5625	0.105	5.360	0.000	0.357	0.768
**Weekend**	0.3271	0.151	2.161	0.031	0.030	0.624
**Income Above $80,820**	-0.7484	0.123	-6.081	0.000	-0.990	-0.507
**More than 55.5% White**	0.6949	0.138	5.044	0.000	0.425	0.965

Table S15: OLS Regression Results - Distances Per Image. This table contains regression results for a regression containing ALL distances, not just those under 9 ft.
